# Preventive effects of tea and tea catechins against influenza and acute upper respiratory tract infections: a systematic review and meta-analysis

**DOI:** 10.1007/s00394-021-02681-2

**Published:** 2021-09-22

**Authors:** Mai Umeda, Takeichiro Tominaga, Kazuya Kozuma, Hidefumi Kitazawa, Daisuke Furushima, Masanobu Hibi, Hiroshi Yamada

**Affiliations:** 1 Biological Science Research Laboratories, Kao Corporation, 2-1-3 Bunka, Sumida-ku, Tokyo 131-8501 Japan; 2 Department of Drug Evaluation and Informatics, Graduate School of Pharmaceutical Sciences, University of Shizuoka, Shizuoka, 422-8526 Japan; 3 Health and Wellness Products Research Laboratories, Kao Corporation, 2-1-3 Bunka, Sumida-ku, Tokyo 131-8501 Japan

**Keywords:** Epigallocatechin gallate, Gargle, Green tea, Non-pharmaceutical intervention, Respiratory virus infection, Tea beverage

## Abstract

**Purpose:**

Gargling with tea has protective effects against influenza infection and upper respiratory tract infection (URTI). To evaluate if tea and tea catechin consumption has the same protective effects as gargling with tea, we performed a systematic review and meta-analysis.

**Methods:**

We performed a comprehensive literature search using the PubMed, Cochrane Library, Web of Science, and Ichu-shi Web databases. The search provided six randomized controlled trials (RCTs) and four prospective cohort studies (*n* = 3748). The quality of each trial or study was evaluated according to the Cochrane risk-of-bias tool or Newcastle–Ottawa Scale. We collected data from publications meeting the search criteria and conducted a meta-analysis of the effect of tea gargling and tea catechin consumption for preventing URTI using a random effects model.

**Results:**

Tea gargling and tea catechin consumption had significant preventive effects against URTI (risk ratio [RR] = 0.74, 95% confidence interval [CI] 0.64–0.87). In sub-analyses, a significant preventive effect was observed by study type (prospective cohort study: RR = 0.67, 95% CI 0.50–0.91; RCT: RR = 0.79, 95% CI 0.66–0.94) and disease type (influenza: RR = 0.69, 95% CI 0.58–0.84; acute URTI: RR = 0.78, 95% CI 0.62–0.98). Both gargling with tea and consuming tea catechins effectively protected against URTI (tea and tea catechins consumption: RR = 0.68, 95% CI 0.52–0.87; tea gargling: RR = 0.83, 95% CI 0.72–0.96).

**Conclusion:**

Our findings suggest that tea gargling and tea catechin consumption may have preventive effects against influenza infection and URTI. The potential effectiveness of these actions as non-pharmaceutical interventions, however, requires further investigation.

## Introduction

Recent pandemics involving influenza [1], severe acute respiratory syndrome-coronavirus (SARS-CoV) [2], and SARS-CoV-2 (COVID-19) [3] have substantially increased global interest in preventive measures against infectious diseases. Given the unpredictable nature of influenza virus, coronavirus, and other respiratory infection virus pandemics, measures aimed at reducing their impact are urgently needed. Non-pharmaceutical interventions (NPIs) are commonly applied in many countries and might be effective in the early stages of viral infection epidemics and pandemics. By reducing the severity early on in epidemics and pandemics, and delaying their peaks, NPIs can decrease the total number of infections and severe cases [4, 5].

NPIs aimed at reducing the transmission of respiratory infections caused by viruses such as influenza include washing hands, wearing masks, physical distancing, and gargling. Although several randomized controlled trials (RCTs) have been performed to evaluate the efficacy of various NPIs, the study quality and intervention adherence have not been adequately assessed [6–10]. Experimental evidence and large-scale RCTs demonstrating the efficacy of hand washing [11] led the World Health Organization (WHO) to consistently recommend this NPI for infectious upper respiratory disease prevention [5]. On the other hand, the evidence from RCTs regarding the effects of wearing masks is limited [10, 12–14]. According to the WHO, masks represent a form of source control and are only recommended for non-infected people during an influenza pandemic [5]. For COVID-19, however, mask wearing by asymptomatic infected people might help prevent the spread of infection, and therefore masks are recommended when social distancing is difficult (e.g., in public transportation) in regions experiencing community-acquired infection [15]. Limited evidence for the effects of gargling and mouthwash to reduce respiratory virus infection has been published, and thus the efficacy of these measures is not well accepted [16]. While these NPIs are potentially promising public health interventions, validation of their effects is needed. Moreover, combining these interventions might strengthen their preventive effects, thereby reducing the severity and delaying the peak of epidemics (e.g., influenza) in the early stages.

Green tea is traditionally consumed in East Asia, but in recent decades it has gained wide popularity around the world [17]. Catechins, which are found in tea leaves (*Camellia sinensis*), are compound mixtures classified as flavanols and include epigallocatechin gallate (EGCg), which has anti-viral effects in vitro [18–21]. Catechins, particularly EGCg, inhibit influenza virus replication in vitro, suggesting that they have a direct anti-viral effect [22]. Because EGCg acts on a viral membrane protein at an early stage of infection, e.g., by inhibiting adsorption, penetration, and membrane fusion, and is present in a common beverage that is available worldwide, it represents an easily accessible NPI against viral respiratory infections. A meta-analysis of data from 3 RCTs and 2 prospective cohort studies by Ide et al. [23] demonstrated that tea gargling has preventive effects against influenza infection (random effects model: risk ratio [RR] = 0.71, 95% confidence interval [CI] 0.56–0.91). Few countries have a custom of gargling, however, and thus widespread implementation of this NPI may be difficult to achieve. Further, it is not clear whether the consumption of tea and tea catechins also has preventive effects against influenza and acute upper respiratory tract infections (URTI). Rowe et al. [24] reported that consuming catechin capsules reduces the incidence of influenza-like symptoms by 32.1%, demonstrating their effectiveness. Similarly, a recent RCT by Furushima et al. [25] demonstrated a significant preventive effect against acute URTI of daily consumption of a tea catechin-containing drink for 12 weeks (hazard ratio = 0.46, 95% CI 0.23–0.95). Although these RCTs suggest that tea catechin consumption has preventive effects against influenza infection and/or acute URTI, comprehensive evidence supporting the effectiveness of tea gargling and tea catechin consumption for preventing viral respiratory infections has not yet been presented.

The present study aimed to evaluate whether gargling or consuming tea and tea catechins has preventive effects against respiratory infections, especially influenza infection and acute URTI, by conducting a systematic review and meta-analysis. As a secondary objective, we assessed the difference in the effectiveness of the interventions by study type, disease type, and tea and tea catechin consumption method.

## Methods

We collected, evaluated, and analyzed published RCTs and prospective cohort studies that quantitatively evaluated the effects of gargling or consuming tea or tea catechins to reduce the risk of viral respiratory infection (e.g., influenza infection and acute URTI), without placing any limits on the subject population.

### Literature search

We first performed a comprehensive literature search using the PubMed, Cochrane Library, Web of Science, and Ichu-shi Web databases, without limiting the language or region. The following search string was used for the PubMed search: (“catechin” OR “tea”) AND (“influenza” OR “upper respiratory tract” OR “common cold”). We also included preprints (bioRxiv, medRxiv) in the search.

### Data collection and quality assessment

For all of the publications included in the analysis, we collected information on the authors, publication date, journal, study design, subject population, place of research, intervention details, randomization method, results, conclusion, and study limitations. To evaluate the risk of bias within an RCT and between RCTs, we used the Cochrane risk-of-bias tool (RoB2.0 tool) [26]. To evaluate the risk of bias in prospective cohort studies, we used the Newcastle–Ottawa Scale [27]. The quality of each study was assessed independently by two of the authors (MU and TT), and in the case of any disagreement regarding the source of potential bias, two other authors were consulted (KK and DF). The present study is a meta-analysis using data from previously published studies, and thus additional informed consent and ethics committee approval were deemed unnecessary.

### Outcome

In accordance with the definition set forth by the WHO, influenza cases were those that were confirmed by laboratory examination (definitive diagnosis based on immunochromatography). Acute URTI cases were those identified on the basis of subjective reports of clinical symptoms, including both influenza-like symptoms and acute URTI symptoms.

### Data analysis

A meta-analysis was conducted using data from six RCTs and four prospective cohort studies with a fixed effects model (Mantel–Haenszel method) and a random effects model (DerSimonian and Laird method) [28]. We assessed the evidence independently and in duplicate using the grading of recommendations assessment, development, and evaluation (GRADE) approach [29]. The results are presented as risk ratio and 95% confidence intervals. Forest plots were used to present effect sizes and pooled estimated values, and funnel plots and Egger’s regression analysis [30] were used to assess publication bias. When publication bias was detected, we used the trim and fill method to correct for deviation of the funnel plot and continued re-calculating until the funnel plot was symmetrical with respect to the estimated values for all analyses [31]. Heterogeneity was evaluated using the *I*^2^ statistic and *Q* test [32]. Heterogeneity was considered high when *I*^2^ > 50% and *p* < 0.1 in the *Q* test. When heterogeneity was high, we determined the reason by performing sensitivity analyses. We conducted a sensitivity analysis to eliminate the effect of inherent methodologic limitations such as observations with a very low number of incidents in the included studies. In the sensitivity analysis, the studies reporting a larger number were compared with the studies reporting a smaller number of incidences (i.e., *n* = 10 or 20). For all statistical analyses, including those for sub-group analyses, we used R version 3.6.1 for Windows with the ‘metafor’ package (The R Foundation for Statistical Computing, Vienna, Austria) [33] and EZR [34].

## Results

### Characteristics of included studies and quality assessment

**Fig. 1:**
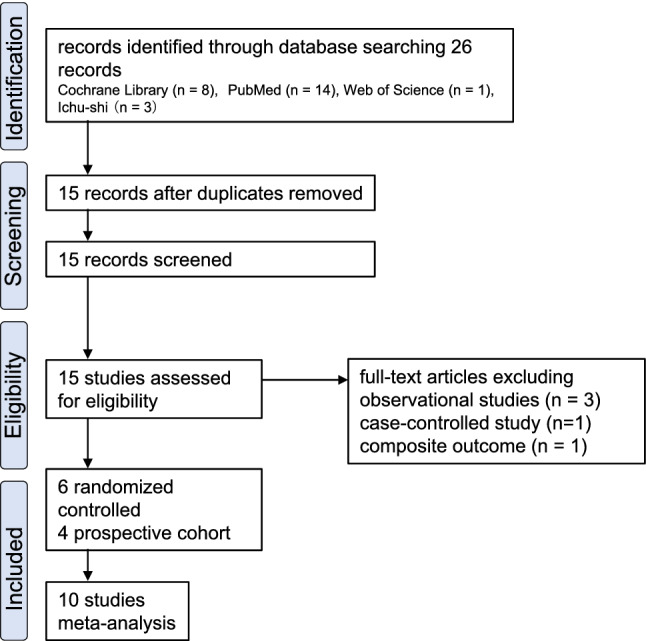
Flow diagram for study selection

The search identified 26 peer-reviewed original publications, 15 of which remained after removing duplicates (Fig. 1). Of these 15 publications, we excluded 3 observational studies [35–37] and 1 case-controlled study [38] that were inherently highly biased and 1 sub-analysis study [39]. Therefore, 6 RCTs and 4 prospective cohort studies were included in the meta-analysis (Table 1) [24, 25, 40–47]. Data were extracted for 6634 subjects, including healthcare facility workers, healthy adults in the community and workplace, and students in junior high school and high school settings. Based on the descriptions in the publications, the subjects were males and females ranging in age from 0 to 83 years [47, 48].

**Table 1:** Study characteristics

Author (year of publication)	Study design	Primary outcome	Country	Setting	Mode of intervention	Population (*n*)	Duration (months)	Intervention (tea or tea catechins: gargling/consumption)	Control
Iwata (1997)	Prospective cohort	Flu	Japan	Workplace	Gargle	297	5	Black tea extract (0.5 w/v%) including EGCg, the aflavin digallate gargling 2 times/day	No intervention
Yamada (2006)	Prospective cohort	Flu	Japan	Healthcare facility	Gargle	124	3	200 μg/ml catechins, 60% of catechins comprise EGCg gargling 3 times/day	Control solution gargling 3 times/day
Yamada (2007)	RCT	Flu	Japan	General community	Gargle	404	3	400 μg/ml catechins, 59.3% of catechins comprise EGCg, 15.1% ECg gargling 3 times/day	Tap water gargling 3 times/day
Rowe (2007)	RCT	URTI	USA	General community	Capsule	124	3	*Camellia sinensis* formulation capsules including L-theanine and EGCg2 capsules/day	Placebo capsule 2 capsules/ day
Matsumoto (2011)	RCT	URTI	Japan	Healthcare facility	Capsule	197	5	63 mg catechins including 45 mg EGCg and 35 mg theanine6 capsules/day	Placebo capsule 6 capsules/day
Yoshioka (2013)	Prospective cohort	Flu	Japan	Junior high school	Drink	236	1–3	Goishi tea (0.4 w/v%)daily consumption	No intervention
Toyoizumi (2013)	RCT	Flu	Japan	High school	Gargle	308	3	56 mg/dL catechins, including 18% EGCg gargling 3 times/day	Water gargling 3 times/day
Ide (2014)	RCT	Flu	Japan	High school	Gargle	757	3	37 ± 0.2 mg/dL catechins, including 18% EGCg gargling 3 times/day	Tap water gargling 3 times/day
Delabre (2015)	Prospective cohort	Flu	France	General community	Drink	1121	2	Green tea or black tea Minimum of 2 times a week	No intervention
Furushima (2020)	RCT	URTI	Japan	Healthcare facility	Drink	270	3	57 mg catechins, including 20 mg EGCg, high-catechins: 3 times/day low-catechins: once/day	Placebo drink once/day

*ECg* epicatechin gallate; *EGCg* epigallocatechin gallate; *Flu* influenza; *RCT* randomized controlled trial; *URTI* upper respiratory tract infection

The overall scientific quality of the four prospective studies, three that were conducted in Japan and one that was conducted in France (CoPanFlu-France cohort [48]) was evaluated with the Newcastle–Ottawa Scale. Five stars were assigned to Iwata et al. [40], eight stars to Yamada et al. [41], seven stars to Yoshioka et al. [42], and eight stars to Delabre et al. [47] (Table 2). For these four studies, we selected exposed cohorts from populations that had a custom of gargling black tea and green tea, consumption of black tea and green tea, and those who consumed Goishi tea, a local type of green tea produced in the Kochi prefecture in Japan.

**Table 2:** Analysis of risk-of-bias using the Newcastle–Ottawa scale

Study	Design	Selection				Comparability	Outcome			Total
		1	2	3	4		1	2	3	
Iwata (1997)	Prospective cohort		◆		◆		◆	◆	◆	5
Yamada (2006)	Prospective cohort	◆	◆	◆	◆	◆	◆	◆	◆	8
Yoshioka (2013)	Prospective cohort	◆	◆	◆	◆		◆	◆	◆	7
Delabre (2015)	Prospective cohort	◆	◆	◆	◆	◆	◆	◆	◆	8

**Fig. 2:**
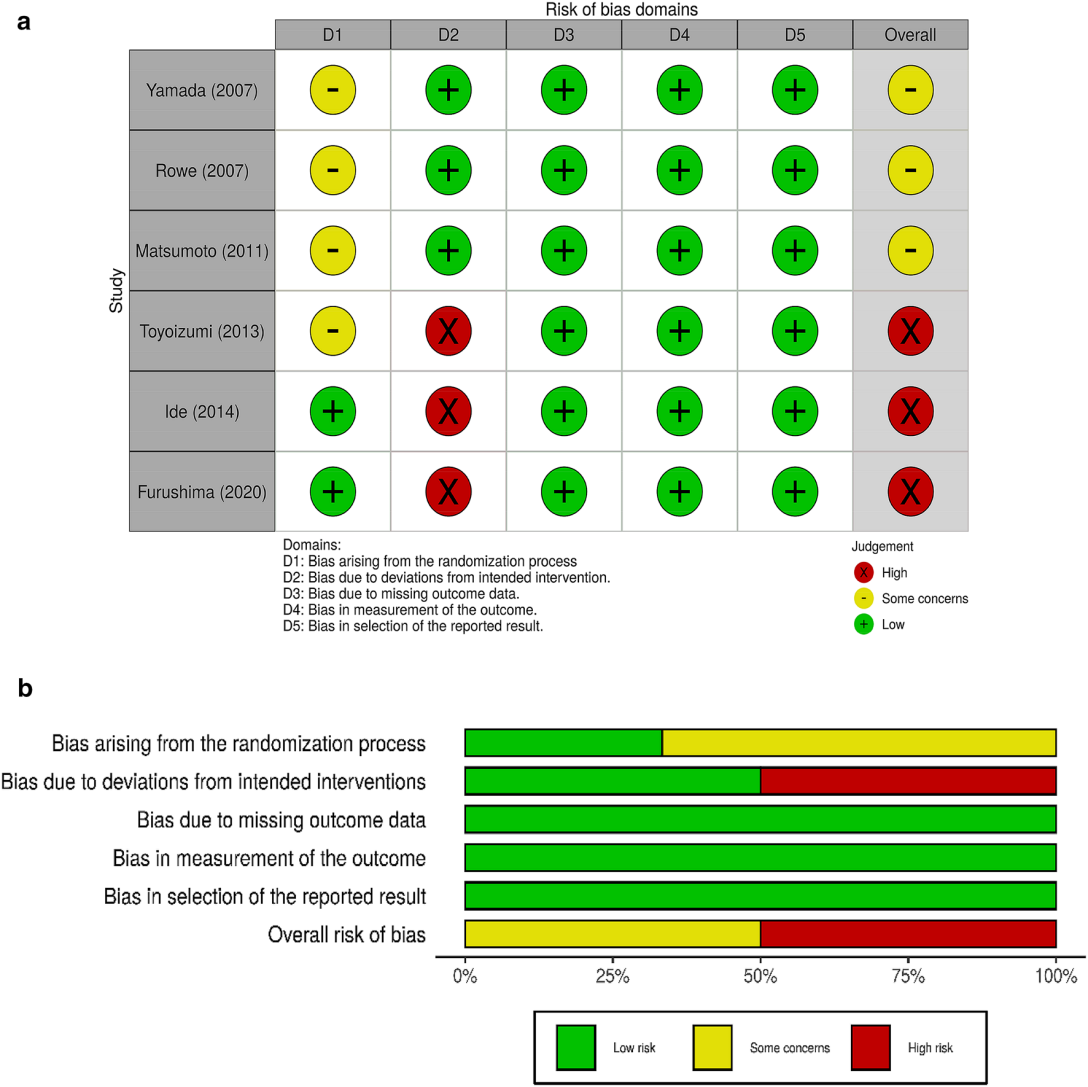
Risk-of-bias assessment of the RCTs. **a** Traffic light plots of domain-level judgements for each individual result. **b** Weighted bar plots of the distribution of risk-of-bias judgements within each bias domain

The six RCTs were assessed using the Cochrane risk-of-bias tool (RoB2.0 tool). Three of the RCTs, i.e., Yamada et al. [43], Rowe et al. [24], and Matsumoto et al. [44], were evaluated overall as having some concerns due to the lack of information regarding the randomization process (Fig. 2). The three remaining RCTs used the open label method (Toyoizumi et al. [45], Ide et al. [46]) or single blind method (Furushima et al. [25]) and were evaluated overall as having a high risk of bias. Interventions in the evaluated RCTs included consumption of green tea extracts (catechin solution for two RCTs, and catechin-containing capsules for two RCTs) or bottled green tea (two RCTs), with placebo (four RCTs) or water (two RCTs) as the control group. Three of the RCTs involved an analysis based on the intent-to-treat principle, and the remaining three involved an analysis based on a full analysis set or per protocol set. Five of the RCTs were conducted in Japan, and the remaining RCT was conducted in Florida in the United States.

### Overall analysis

**Fig. 3:**
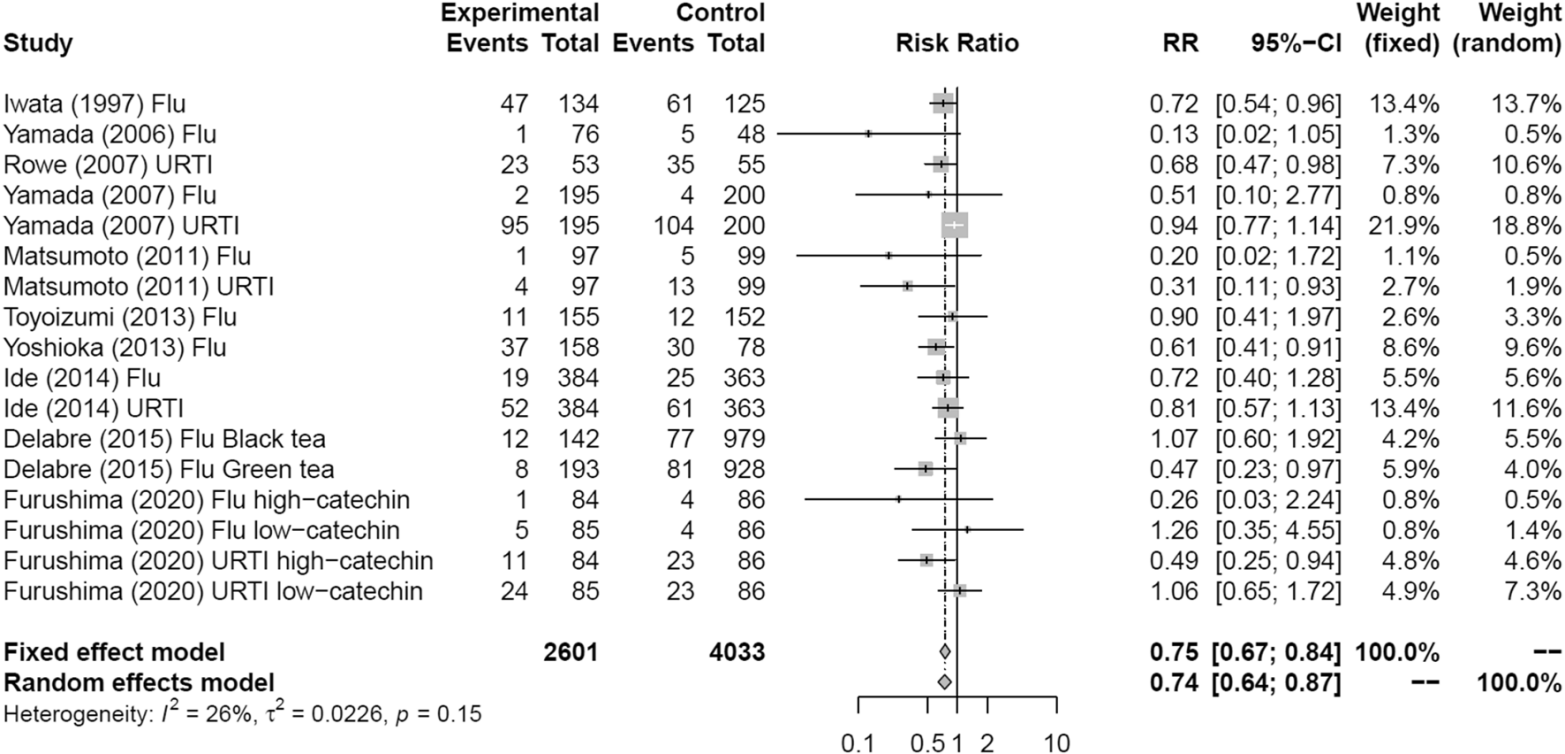
Forest plot of tea or tea catechins versus control on influenza infection and acute upper respiratory tract infection. *CI* confidence interval; *Flu* influenza infection; *URTI* upper respiratory tract infection

Figure 3 shows a forest plot of risk ratios determined from the six RCTs and four prospective cohort studies that examined the preventive effects of tea gargling and tea catechin consumption against both influenza infection and acute URTI. Compared with the control groups (i.e., no intervention, placebo or water gargling), the rates of influenza infection and acute URTI in the intervention groups (tea catechin consumption or tea gargling) were significantly reduced (random effects model: RR = 0.74, 95% CI 0.64–0.87).

**Fig. 4:**
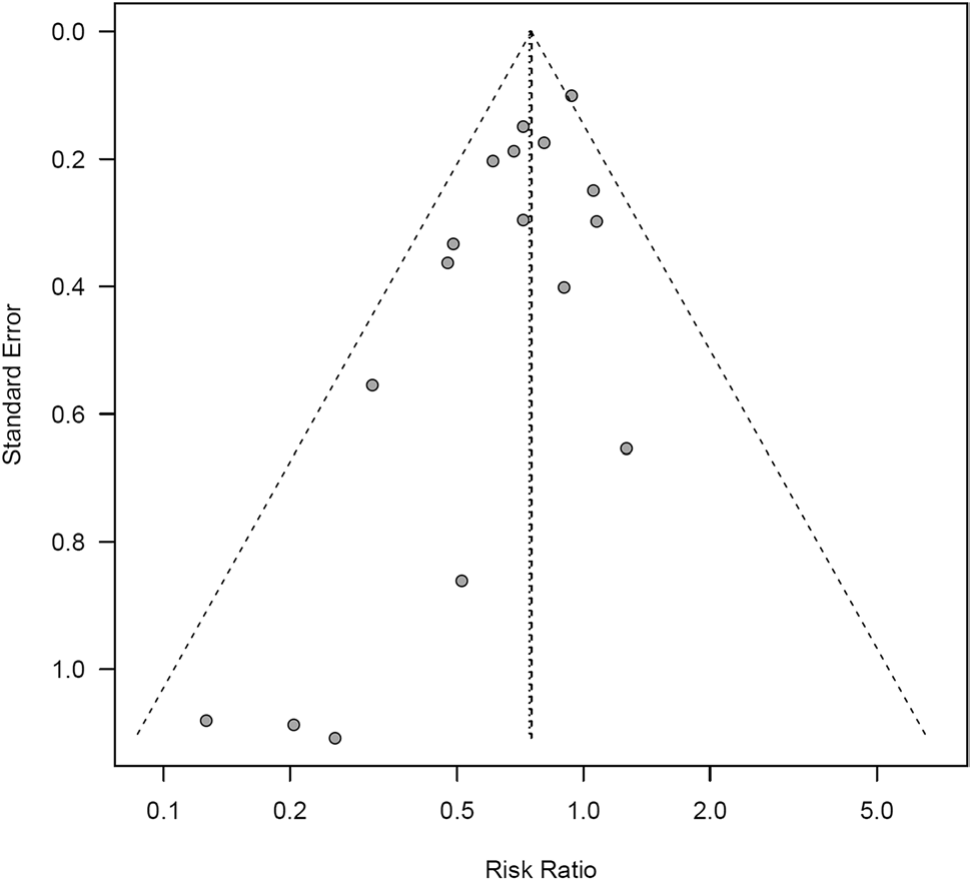
Funnel plot of tea or tea catechins versus control on influenza infection and acute upper respiratory tract infection

The *Q* test and *I*^2^ statistic demonstrated low heterogeneity of the studies (*I*^2^ = 26.3%, *Q* = 21.70, *τ*^2^ = 0.0226, *p* = 0.15). Figure 4 shows a funnel plot for publication bias. The potential bias, as reflected by the empty space in the bottom right region of the graph, was suggestive of bias in the small-scale studies. Egger’s regression analysis revealed significant differences (*p* = 0.01). After correcting for the bias using the trim and fill method, five data points were corrected, but the result remained the same, with a significantly reduced rate of influenza infection and acute URTI in the intervention groups (random effects model: RR = 0.79, 95% CI 0.67–0.93).

### Sub-group analysis by RCTs or prospective cohort studies

**Fig. 5:**
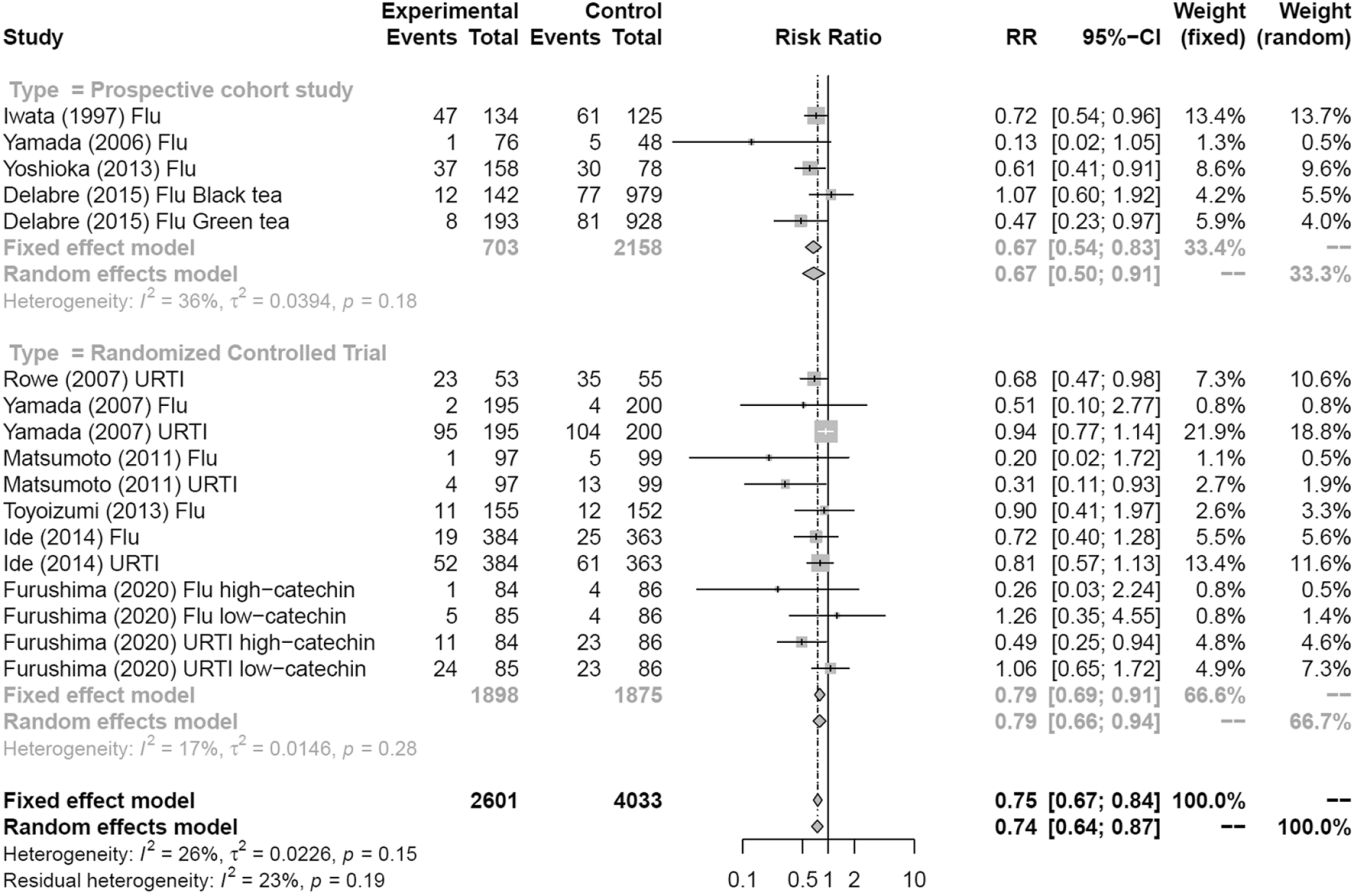
Forest plots of meta-analysis results of sub-analysis by study type: tea and tea catechins versus control on RCTs and prospective cohort studies. *CI* confidence interval; *Flu* influenza infection; *RCTs* randomized controlled trials; *URTI* upper respiratory tract infection

Figure 5 shows a forest plot of risk ratios determined from the six RCTs and four prospective cohort studies that examined the preventive effects of tea gargling and tea catechin consumption against influenza infection and acute URTI. Four prospective cohort studies showed a significantly reduced rate of influenza infection compared with the control groups (no intervention, gargling with placebo or water, or placebo consumption; random effects model: RR = 0.67, 95% CI 0.50–0.91). The same analysis was performed for the six RCTs that examined the preventive effects of tea gargling and tea catechin consumption against influenza infection and acute URTI. These interventions also led to a significantly reduced rate of influenza infection and acute URTI compared with the control groups (random effects model: RR = 0.79, 95% CI 0.66–0.94).

### Sub-group analysis by influenza infection or acute URTI

**Fig. 6:**
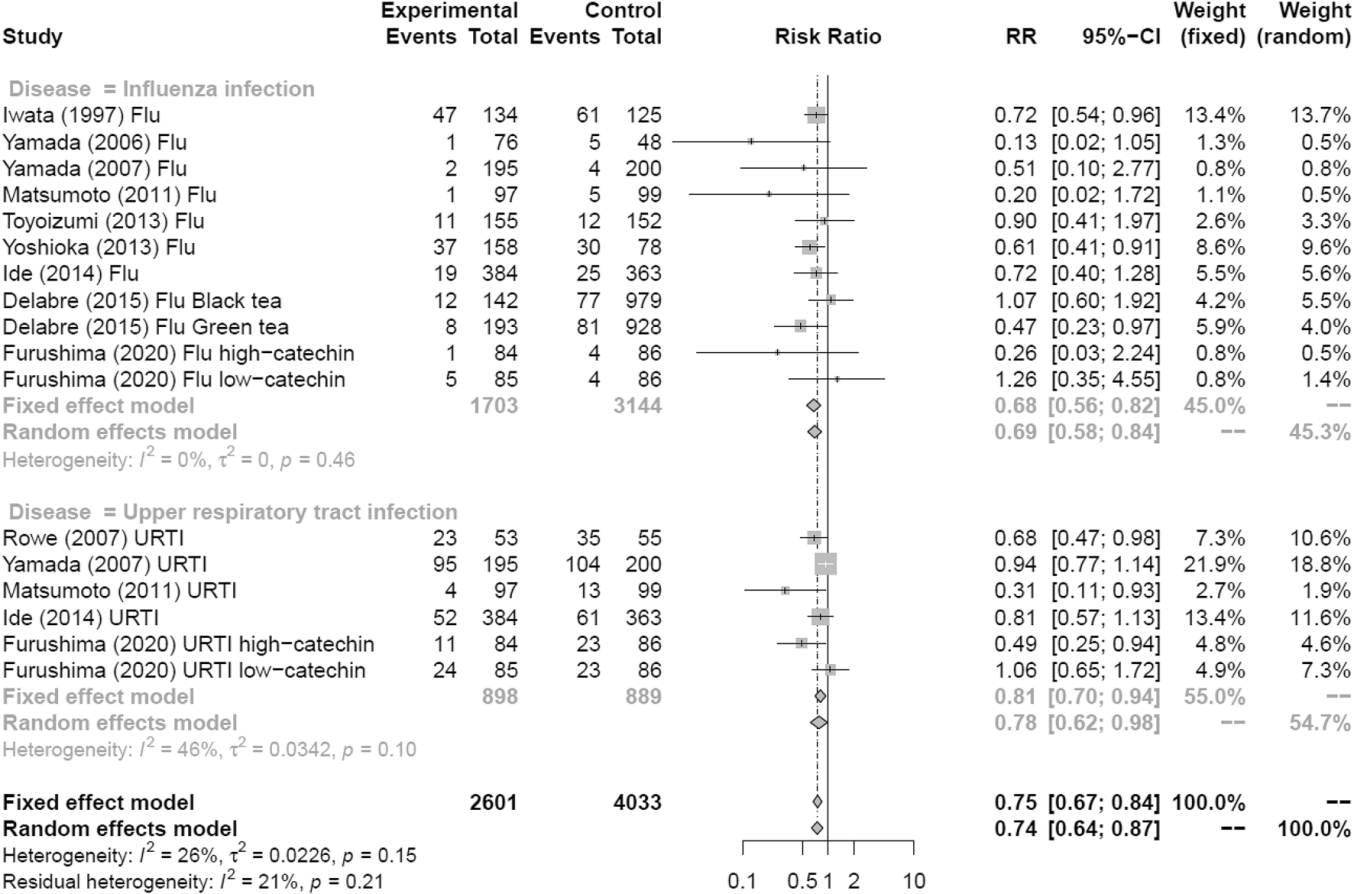
Forest plots of meta-analysis results of sub-analysis by disease type: tea and tea catechins versus control on acute upper respiratory tract infection and influenza infection. *CI* confidence interval; *Flu* influenza infection; *URTI* upper respiratory tract infection

Figure 6 shows a forest plot of risk ratios determined from the five RCTs and four prospective cohort studies that examined the preventive effects of tea gargling and tea catechin consumption against influenza infection. These interventions significantly reduced the rate of influenza infection compared with the control groups (no intervention, gargling with placebo or water, or placebo consumption; random effects model: RR = 0.69, 95% CI 0.58–0.84). The same analysis was performed for the five RCTs that examined the preventive effects of tea gargling and tea catechin consumption against acute URTI. These interventions also significantly reduced the rate of acute URTI compared with the control groups (random effects model: RR = 0.78, 95% CI 0.62–0.98).

### Sub-group analysis by tea catechin consumption or tea gargling

**Fig. 7:**
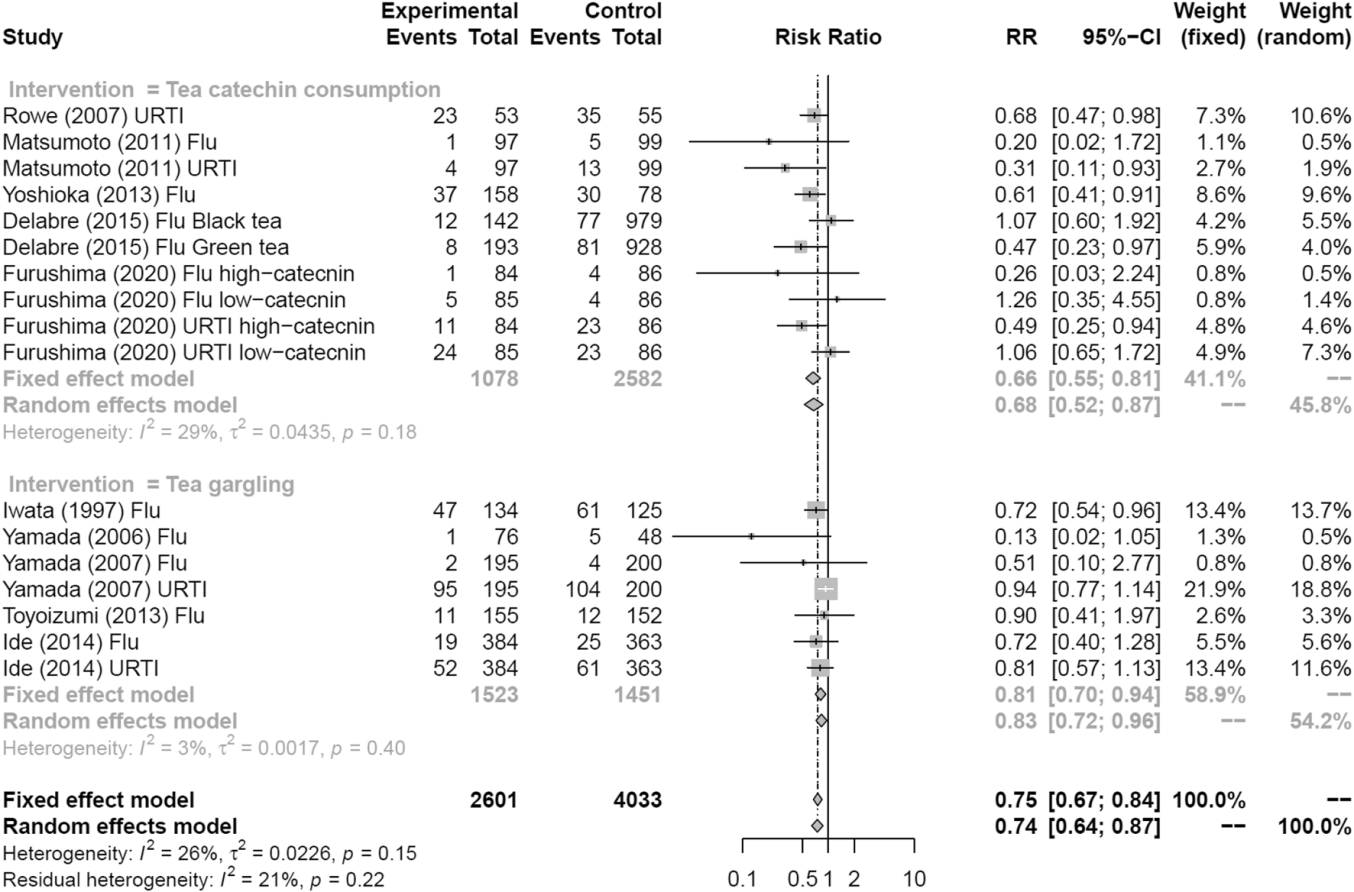
Forest plots of meta-analysis results of sub-analysis by method of intervention: tea catechin consumption and tea gargling versus control on influenza infection and acute upper respiratory tract infection. *CI* confidence interval; *Flu* influenza infection; *URTI* upper respiratory tract infection

Figure 7 shows a forest plot of risk ratios determined from the three RCTs and two prospective cohort studies that examined the preventive effects of consuming tea or tea catechins against influenza infection and acute URTI. The tea catechin consumption group had a significantly reduced rate of influenza infection and acute URTI compared with the placebo consumption group (random effects model: RR = 0.68, 95% CI 0.52–0.87). Figure 7 also shows a forest plot of risk ratios determined from the three RCTs and two prospective cohort studies that examined the preventive effects of tea gargling against influenza infection and acute URTI. The tea gargling group had a significantly reduced rate of influenza infection and acute URTI compared with the group that gargled placebo or water (random effects model: RR = 0.83, 95% CI 0.72–0.96).

**Fig. 8:**
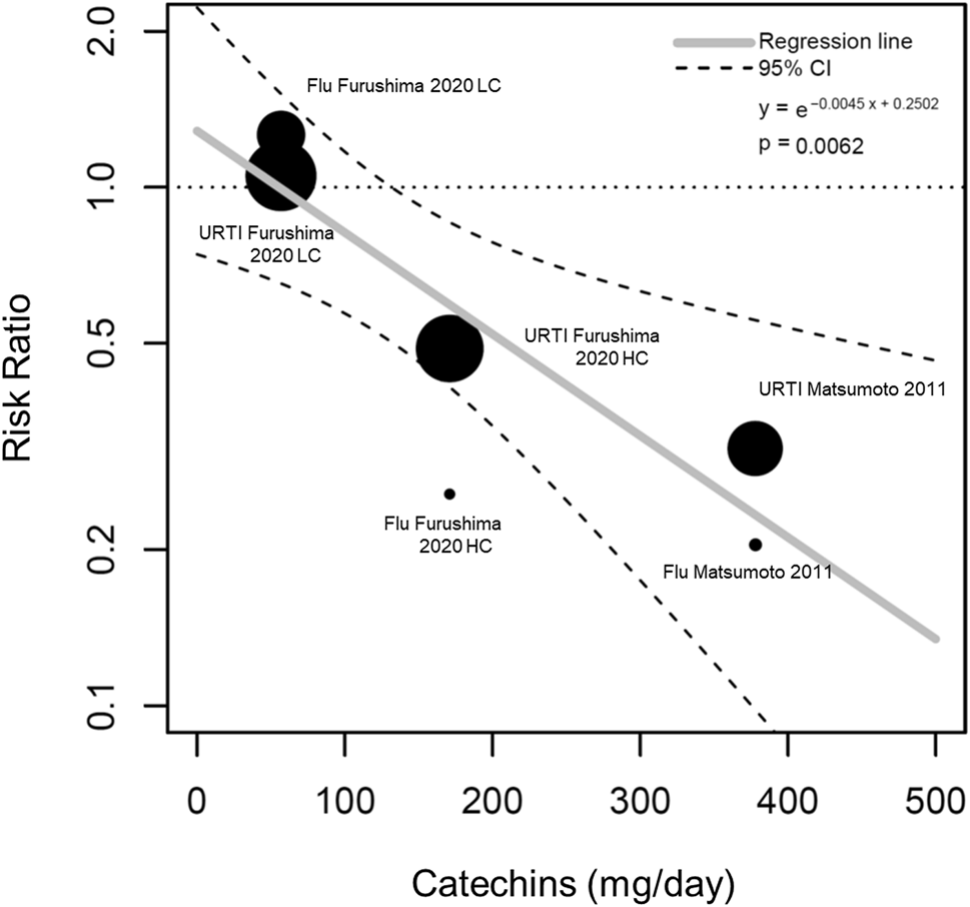
Dose dependency of catechin effects using meta-regression. *CI* confidence interval; *Flu* influenza infection; *URTI* upper respiratory tract infection; *HC* high-catechin group; *LC* low catechin group

For published RCTs examining the preventive effects of tea catechin consumption on influenza infection and acute URTI, descriptions of the daily amount of tea catechin consumption were provided, which allowed us to conduct a meta-analysis on the dose-dependency of tea catechins (Fig. 8). This analysis used data from two studies that clearly described the total amount of daily tea catechin consumption (Furushima et al. [25] and Matsumoto et al. [44]). A significant correlation was detected between the daily amount of tea catechin consumption and risk ratio (*p* < 0.01).

### Sensitivity analysis

**Fig. 9:**
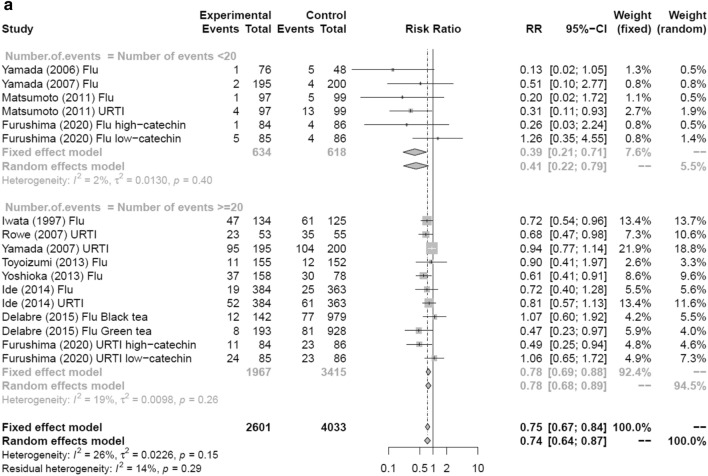

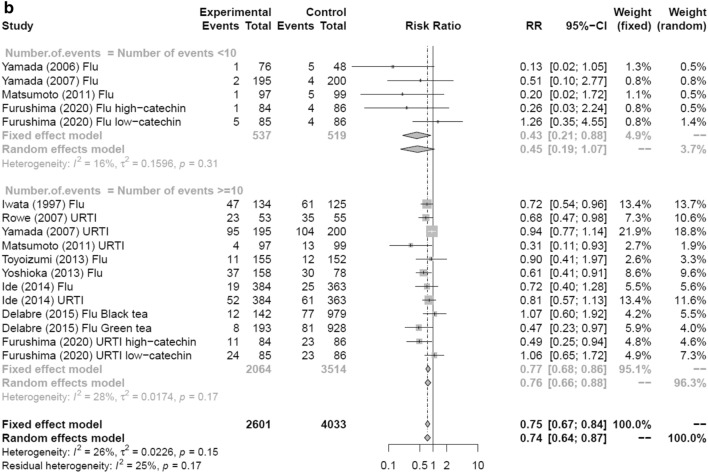
Forest plots of sensitivity analysis by the number of events. **a** Sub-analysis with a cutoff number of 20 events. **b** Sub-analysis with a cutoff number of ten events. *CI* confidence interval; *Flu* influenza infection; *URTI* upper respiratory tract infection

In the sensitivity analysis, pooled trials were performed to examine the preventive effects of tea and tea catechins on influenza infection and UTRI compared with a control group when the cutoff for the number of events during the study was 10 or less, or when the cutoff for the number of events was 20 or less. Figure 9a shows that analysis of studies with at least 20 events revealed an RR = 0.78 (random effects model: 95% CI 0.68–0.89, k = 11, *I*^2^ = 19.1%, *Q* = 12.36, *τ*^2^ = 0.01). Analysis of studies with 20 or fewer events also had an RR = 0.41 (random effects model: 95% CI 0.22–0.79, *k* = 6, *I*^2^ = 1.9%, *Q* = 5.10, *τ*^2^ = 0.01), indicating a significant effect between control groups in both comparisons.

When the cutoff was ten events, in studies with more than ten events, the RR of influenza and URTI with consumption of tea and tea catechins was 0.76 (95% CI 0.66–0.88, *k* = 12, *I*^2^ = 27.9%, *Q* = 15.26, *τ*^2^ = 0.02), which was significant when compared with the control group (Fig. 9b). When the cutoff was ten or fewer events, the RR = 0.45 (95% CI 0.19–1.07, *k* = 5, *I*^2^ = 15.8%, *Q* = 4.75, *τ*^2^ = 0.16) and there was no significant effect compared with the control group. A comparison between studies with more than ten events and fewer than ten events revealed no significant difference between them (*p* = 0.24).

## Discussion

The present meta-analysis including data from 3748 subjects with viral respiratory infections from 6 RCTs and 4 prospective cohort studies revealed significant preventive effects of tea gargling and tea catechin consumption against influenza infection and acute URTI compared with controls (RR = 0.74, 95% CI 0.64–0.87). This study represents the first comprehensive analysis of the preventive effects of tea gargling and tea catechin consumption against these viral infections. The strength of our study lies in the complete adherence to systematic review methods, including the two-tiered screening process of publication titles and abstracts by independent researchers, evaluation of the quality of each study, evaluation of risk of bias, and no limitation regarding the language used in the publications. All cohort studies and RCTs were well designed, with subject ages ranging from 0 to 83 years. Subjects included healthcare workers, junior high school and high school students, and people recruited from the general population, suggesting that the effects of the interventions are unlikely to be dependent on a particular age group or setting.

The WHO announced a worldwide recommendation for the introduction of NPIs in response to the COVID-19 pandemic in 2020 [49]. According to the latest information from the CDC, the primary route of transmission of SARS-CoV-2 is through exposure to respiratory fluids containing the infectious virus. [50]. Therefore, NPIs were introduced with the aim of significantly reducing the frequency of contact and slowing down the spread of the virus in the population, including countries, regions, workplaces, and schools [51]. In a recent meta-analysis of the preventive effects of face masks against viral respiratory infections, however, influenza-like symptoms (acute URTI) were not significantly reduced by the use of face masks alone or in combination with handwashing (face mask alone: pooled effect size, − 0.17, 95% CI − 0.43–0.10, *p* = 0.23; combination of face mask and handwashing: pooled effect size, − 0.09, 95% CI − 0.58–0.40, *p* = 0.71) [52]. While examining the effects of various combinations of NPIs could provide important insight into inhibiting the spread of viral respiratory infections, our findings suggest that tea gargling and tea catechin consumption may present a relatively easy and effective way to achieve this goal.

To our knowledge, the present study is the first to quantify the protective effects of tea gargling and tea catechin consumption against influenza infection and URTI. Specifically, compared with controls, tea gargling and tea catechin consumption exhibited significant preventive effects against influenza infection and acute URTI. Viruses that cause acute URTI include rhinovirus, coronavirus, influenza virus, respiratory syncytial virus, adenovirus, and others [53, 54]. Given the evidence that EGCg, a main component of tea catechins, provides effective protection against influenza virus [55] and adenovirus [56], it may also protect against respiratory infections caused by other viruses.

In the intervention method analyses, consumption of tea or tea catechins through catechin-containing drinks or catechin-containing capsules prevented both influenza infection and acute URTI (RR = 0.68, 95% CI 0.52–0.87). Tea gargling similarly provided effective protection against influenza infection and acute URTI (RR = 0.83, 95% CI 0.72–0.96). The preventive effect of tea gargling in the present study is consistent with findings of the meta-analysis performed by Ide et al. [23], confirming the robustness of our study. EGCg and epigallocatechin (EGC) bind to the hemagglutinin spike on the viral surface and neuraminidase to inhibit attachment of the virus to the cell surface, thereby preventing influenza infection [57], and both EGCg and EGC inhibit viral RNA synthesis and thus proliferation by targeting the viral RNA polymerase [56]. A recent SARS-CoV-2 docking simulation study also found that EGCg, EGC, and other catechins have strong binding affinity for the main protease of SARS-CoV-2 [58]. Together, these studies suggest that the effects observed with tea gargling and tea or tea catechin consumption potentially reflect physical removal of the virus via the binding of catechins to the virus, or that catechins inhibit the attachment and proliferation of the virus on the surface of the upper respiratory tract. Consumed tea or tea catechins may also form a barrier in the pharynx and inhibit the attachment to and proliferation of viruses in the upper respiratory tract, as well as physically flush viruses attached to the surface of the upper respiratory tract into the stomach. Oral administration of an EGC/EGCg mixture in mice increases IgA production in the intestinal mucosa and promotes mucosal immunity [59]. Thus, EGCg or its metabolites from catechin-containing drinks and catechin-containing capsules may have preventive effects by enhancing immune function.

The preventive effects described in the present study likely derive mainly from the effects of tea or tea catechins. Some of the studies we assessed provided clear descriptions regarding the amounts of tea catechin consumed. This allowed us to conduct a meta-analysis on the dose-dependency of the catechin effect, which revealed a significant correlation between the risk ratio and the total daily amount of catechins consumed (*p* < 0.01). Specifically, the larger the amount of daily catechin consumption, the stronger the preventive effect against the spread of viral respiratory infection. Furthermore, as reported by Furushima et al. [25], drinking a tea catechin-containing drink three times a day (high-catechin group) had a stronger preventive effect than drinking it once a day. Similarly, Matsumoto et al. [44] reported strong effects of consuming large amounts of catechins per day (catechin-containing capsule six times a day). These studies suggest that the frequency of catechin consumption also influences its preventive effects against viral infection.

In addition, because the present study surveyed literature that included smaller sized studies, we conducted a sensitivity analysis based on the number of events. When comparing only the studies with ten or fewer events, no significant difference was observed compared with the control group. In the analysis excluding the studies with fewer than ten events, which are less reliable, a significant preventive effect of catechin consumption or gargling was observed compared with the control group. The results showed that the consumption or gargling of tea and tea catechins had a significant preventive effect compared with the control groups when comparing reliable studies with more than ten events.

## Limitations

This study has some limitations. First, comprehensive analysis of RCTs and prospective cohort studies, including one study in the USA, one study in France, and the rest in Japan, may have a potential regional bias with respect to the effects of tea and tea catechins on influenza infection and acute URTI. The majority of the trials evaluated (80%) were conducted in Japan, while only 20% of trials were conducted in the USA and France. The French trial was a large prospective cohort study, however, and 35% of the cases were from trials conducted in the USA and France. Although all published studies were collected and analyzed, this study was conducted in Japan, potentially biasing it toward East Asia, especially Japan. Our findings may reflect the fact that the custom of tea gargling and green tea consumption is unique to East Asia, including Japan, and thus the generalizability of our results may be limited. Future studies should consider further advances in our understanding of detection capabilities when estimating the preventive effects of tea against infectious diseases. Second, the funnel plot analysis suggested a publication bias, which could have overestimated the preventive effects of tea gargling and tea catechin consumption against influenza infection and acute URTI. Although five points were corrected for bias using the trim and fill method, however, the rate of influenza infection and acute URTI remained significantly reduced. Third, awareness regarding the prevention of influenza infection and acute URTI during an epidemic might have been increased by participation in the RCTs. Future large-scale studies to examine the preventive effects of consuming tea or its components against viral respiratory infections are warranted.

## Conclusion

We present the results of a systematic review and meta-analysis of data of 3748 participants collected from 6 RCTs and 4 prospective cohort studies indicating that tea gargling and tea catechin consumption have preventive effects against viral respiratory infections, such as influenza infection and acute URTI. Given the potential biases in the present study, as well as the fact that only four of the studies evaluated the effects of tea catechin consumption and six of the studies evaluated the effects of tea gargling, a large-scale RCT would help validate the preventive effects of tea and tea catechins on viral respiratory infections. A dose-dependent relationship was observed between the total daily amount of catechin consumption and the preventive effects against infection, suggesting that the preventive effects are likely due to the catechins. Our findings suggest that incorporating tea catechin consumption and/or tea gargling into the daily routine may be effective NPIs for preventing viral respiratory infections, but further large-scale studies are needed to confirm these findings.

## References

[1] Swerdlow DL, Finelli L, Bridges CB (2011). 2009 H1N1 influenza pandemic: field and epidemiologic investigations in the United States at the start of the first pandemic of the 21st century. Clin Infect Dis.

[2] Peiris JS, Yuen KY, Osterhaus AD, Stöhr K (2003). The severe acute respiratory syndrome. N Engl J Med.

[3] Ge H, Wang X, Yuan X (2020). The epidemiology and clinical information about COVID-19. Eur J Clin Microbiol Infect Dis.

[4] Aledort JE, Lurie N, Wasserman J, Bozzette SA (2007). Non-pharmaceutical public health interventions for pandemic influenza: an evaluation of the evidence base. BMC Public Health.

[5] World Health Organization (WHO) (2019) Non-pharmaceutical public health measures for mitigating the risk and impact of epidemic and pandemic influenza. https://www.who.int/influenza/publications/public_health_measures/publication/en/. Accessed 1 July 2020

[6] Wong VW, Cowling BJ, Aiello AE (2014). Hand hygiene and risk of influenza virus infections in the community: a systematic review and meta-analysis. Epidemiol Infect.

[7] Moncion K, Young K, Tunis M, Stirling R, Zhao L (2009). Effectiveness of hand hygiene practices in preventing influenza virus infection in the community setting: a systematic review. Can Commun Dis Rep.

[8] Smith SM, Sonego S, Wallen GR, Waterer G, Cheng AC, Thompson P (2015). Use of non-pharmaceutical interventions to reduce the transmission of influenza in adults: a systematic review. Respirology.

[9] Jefferson T, Del Mar C, Dooley L (2010). Physical interventions to interrupt or reduce the spread of respiratory viruses. Cochrane Database Syst Rev.

[10] World Health Organization (WHO) (2019) Non-pharmaceutical public health measures for mitigating the risk and impact of epidemic and pandemic influenza: annex: report of systematic literature reviews. https://apps.who.int/iris/handle/10665/329439. Accessed 1 May 2020

[11] Talaat M, Afifi S, Dueger E (2011). Effects of hand hygiene campaigns on incidence of laboratory-confirmed influenza and absenteeism in schoolchildren, Cairo. Egypt Emerg Infect Dis.

[12] Long Y, Hu T, Liu L (2020). Effectiveness of N95 respirators versus surgical masks against influenza: a systematic review and meta-analysis. J Evid Based Med.

[13] Saunders-Hastings P, Crispo JAG, Sikora L, Krewski D (2017). Effectiveness of personal protective measures in reducing pandemic influenza transmission: a systematic review and meta-analysis. Epidemics.

[14] Bin-Reza F, Lopez Chavarrias V, Nicoll A, Chamberland ME (2012). The use of masks and respirators to prevent transmission of influenza: a systematic review of the scientific evidence. Influenza Other Respir Viruses.

[15] World Health Organization (WHO) (2020) Coronavirus disease (COVID-19) advice for the public: When and how to use masks. https://www.who.int/emergencies/diseases/novel-coronavirus-2019/advice-for-public. Accessed 1 July 2020

[16] Satomura K, Kitamura T, Kawamura T, Great Cold Investigators-I (2005). Prevention of upper respiratory tract infections by gargling: a randomized trial. Am J Prev Med.

[17] Graham HN (1992). Green tea composition, consumption, and polyphenol chemistry. Prev Med.

[18] Lin JK, Lin CL, Liang YC, Lin-Shiau SY, Juan IM (1998). Survey of catechins, gallic acid, and methylxanthines in green, oolong, pu-erh, and black teas. J Agric Food Chem.

[19] Perva-Uzunalić A, Škerget M, Knez Ž, Weinreich B, Otto F, Grüner S (2006). Extraction of active ingredients from green tea (*Camellia sinensis*): extraction efficiency of major catechins and caffeine. Food Chem.

[20] Del Rio D, Stewart AJ, Mullen W (2004). HPLC-MSn analysis of phenolic compounds and purine alkaloids in green and black tea. J Agric Food Chem.

[21] Steinmann J, Buer J, Pietschmann T, Steinmann E (2013). Anti-infective properties of epigallocatechin-3-gallate (EGCG), a component of green tea. Br J Pharmacol.

[22] Kaihatsu K, Yamabe M, Ebara Y (2018). Antiviral mechanism of action of epigallocatechin-3-*O*-gallate and its fatty acid esters. Molecules.

[23] Ide K, Yamada H, Kawasaki Y (2016). Effect of gargling with tea and ingredients of tea on the prevention of influenza infection: a meta-analysis. BMC Public Health.

[24] Rowe CA, Nantz MP, Bukowski JF, Percival SS (2007). Specific formulation of Camellia sinensis prevents cold and flu symptoms and enhances gamma, delta T cell function: a randomized, double-blind, placebo-controlled study. J Am Coll Nutr.

[25] Furushima D, Nishimura T, Takuma N (2020). Prevention of acute upper respiratory infections by consumption of catechins in healthcare workers: a randomized, placebo-controlled trial. Nutrients.

[26] Higgins JPT, Green S (2011) Cochrane Handbook for Systematic Reviews of Interventions, version 5.1.0. https://handbook.cochrane.org/. Accessed 1 July 2020

[27] Wells GA, Shea B, O'Connell D et al (2020) The Newcastle-Ottawa Scale (NOS) for assessing the quality of nonrandomised studies in meta-analyses. http://www.ohri.ca/programs/clinical_epidemiology/oxford.asp. Accessed 1 July 2020

[28] Mantel N, Haenszel W (1959). Statistical aspects of the analysis of data from retrospective studies of disease. J Natl Cancer Inst.

[29] Atkins D, Eccles M, Flottorp S, GRADE Working Group (2004). Grading quality of evidence and strength of recommendations. BMJ.

[30] Egger M, Davey Smith G, Schneider M, Minder C (1997). Bias in meta-analysis detected by a simple, graphical test. BMJ.

[31] Duval S, Tweedie R (2000). Trim and fill: a simple funnel-plot-based method of testing and adjusting for publication bias in meta-analysis. Biometrics.

[32] Higgins JP, Thompson SG (2002). Quantifying heterogeneity in a meta-analysis. Stat Med.

[33] Viechtbauer W (2010). Conducting meta-analyses in R with the metafor package. J Stat Software.

[34] Kanda Y (2013). Investigation of the freely available easy-to-use software EZR for medical statistics. Bone Marrow Transplant.

[35] Noda T, Ojima T, Hayasaka S, Murata C, Hagihara A (2012). Gargling for oral hygiene and the development of fever in childhood: a population study in Japan. J Epidemiol.

[36] Park M, Yamada H, Matsushita K (2011). Green tea consumption is inversely associated with the incidence of influenza infection among schoolchildren in a tea plantation area of Japan. J Nutr.

[37] Nanri A, Nakamoto K, Sakamoto N, Imai T, Mizoue T (2020). Green tea consumption and influenza infection among Japanese employees. Eur J Clin Nutr.

[38] Kondo K, Suzuki K, Washio M, The Pneumonia in Elderly People Study Group (2021). Association between coffee and green tea intake and pneumonia among the Japanese elderly: a case-control study. Sci Rep.

[39] Ide K, Kawasaki Y, Akutagawa M, Yamada H (2017). Effects of green tea gargling on the prevention of influenza infection: an analysis using Bayesian approaches. J Altern Complement Med.

[40] Iwata M, Toda M, Nakayama M (1997). Prophylactic effect of black tea extract as gargle against influenza. Kansenshogaku Zasshi.

[41] Yamada H, Takuma N, Daimon T, Hara Y (2006). Gargling with tea catechin extracts for the prevention of influenza infection in elderly nursing home residents: a prospective clinical study. J Altern Complement Med.

[42] Yoshioka S, Ju-Ngam T, Jobu K (2013). The protective effects of Goishi tea against influenza infection. Jpn Pharmacol Therap.

[43] Yamada H, Daimon T, Matsuda K, Yoshida M, Takuma N, Hara Y (2007). A randomized controlled study on the effects of gargling with tea catechin extracts on the prevention of influenza infection in healthy adults. Jpn J Clin Pharmacol Ther.

[44] Matsumoto K, Yamada H, Takuma N, Niino H, Sagesaka YM (2011). Effects of green tea catechins and theanine on preventing influenza infection among healthcare workers: a randomized controlled trial. BMC Complement Altern Med.

[45] Toyoizumi K, Yamada H, Matsumoto K, Sameshima Y (2013). Gargling with green tea for influenza prophylaxis: a pilot clinical study. Jpn J Clin Pharmacol Ther.

[46] Ide K, Yamada H, Matsushita K (2014). Effects of green tea gargling on the prevention of influenza infection in high school students: a randomized controlled study. PLoS ONE.

[47] Delabre RM, Lapidus N, Salez N, Mansiaux Y, de Lamballerie X, Carrat F (2015). Risk factors of pandemic influenza A/H1N1 in a prospective household cohort in the general population: results from the CoPanFlu-France cohort. Influenza Other Respir Viruses.

[48] Lapidus N, de Lamballerie X, Salez,  (2012). Integrative study of pandemic A/H1N1 influenza infections: design and methods of the CoPanFlu-France cohort. BMC Public Health.

[49] World Health Organization (WHO) (2020) Coronavirus disease (COVID-19) pandemic. https://www.euro.who.int/en/health-topics/health-emergencies/coronavirus-covid-19/novel-coronavirus-2019-ncov. Accessed 1 July 2020

[50] Centers for Disease Control and Prevention (CDC) (2021) Scientific Brief: SARS-CoV-2 Transmission. https://www.cdc.gov/coronavirus/2019-ncov/science/science-briefs/sars-cov-2-transmission.html. Accessed 31 May 2021 34009775

[51] Flaxman S, Mishra S, Gandy A (2020). Estimating the effects of non-pharmaceutical interventions on COVID-19 in Europe. Nature.

[52] MacIntyre CR, Chughtai AA (2020). A rapid systematic review of the efficacy of face masks and respirators against coronaviruses and other respiratory transmissible viruses for the community, healthcare workers and sick patients. Int J Nurs Stud.

[53] Mäkelä MJ, Puhakka T, Ruuskanen O (1998). Viruses and bacteria in the etiology of the common cold. J Clin Microbiol.

[54] Heikkinen T, Järvinen A (2003). The common cold. Lancet.

[55] Song JM, Lee KH, Seong BL (2005). Antiviral effect of catechins in green tea on influenza virus. Antiviral Res.

[56] Weber JM, Ruzindana-Umunyana A, Imbeault L, Sircar S (2003). Inhibition of adenovirus infection and adenain by green tea catechins. Antiviral Res.

[57] Nakayama M, Suzuki K, Toda M, Okubo S, Hara Y, Shimamura T (1993). Inhibition of the infectivity of influenza virus by tea polyphenols. Antiviral Res.

[58] Ghosh R, Chakraborty A, Biswas A, Chowdhuri S (2020). Evaluation of green tea polyphenols as novel corona virus (SARS CoV-2) main protease (Mpro) inhibitors - an in silico docking and molecular dynamics simulation study. J Biomol Struct Dyn.

[59] Monobe M, Ema K, Tokuda Y, Maeda-Yamamoto M (2010). Effect on the epigallocatechin gallate/epigallocatechin ratio in a green tea (*Camellia sinensis* L.) extract of different extraction temperatures and its effect on IgA production in mice. Biosci Biotechnol Biochem.

